# Crystal structure of 2-[4-(methyl­sulfan­yl)quinazolin-2-yl]-1-phenyl­ethanol

**DOI:** 10.1107/S1600536814019990

**Published:** 2014-09-10

**Authors:** Gamal A. El-Hiti, Keith Smith, Amany S. Hegazy, Mansour D. Ajarim, Benson M. Kariuki

**Affiliations:** a Cornea Research Chair, Department of Optometry, College of Applied Medical Sciences, King Saud University, PO Box 10219, Riyadh 11433, Saudi Arabia; b School of Chemistry, Cardiff University, Main Building, Park Place, Cardiff CF10 3AT, Wales; c Criminal Evidence, Ministry of Interior, Riyadh 11632, PO Box 86985, Saudi Arabia

**Keywords:** crystal structure, 4-(methyl­sulfan­yl)quinazoline derivative, hydrogen bonding

## Abstract

In the mol­ecule of the title compound, C_17_H_16_N_2_OS, the almost planar methyl­sulfanylquinazoline group [the methyl C atom deviates by 0.032 (2) Å from the plane through the ring system] forms an inter­planar angle of 76.26 (4)° with the plane of the phenyl group. An intra­molecular O—H⋯N hydrogen bond is present between the quinazoline and hy­droxy groups. In the crystal, mol­ecules are stacked along the *b*-axis direction.

## Related literature   

For the synthesis of 4-(methyl­sulfan­yl)quinazoline derivatives, see: Smith *et al.* (2005*a* ▶,*b* ▶); Leonard & Curtin (1946 ▶); Meerwein *et al.* (1956 ▶). For the crystal structures of related compounds, see: Alshammari *et al.* (2014*a* ▶,*b* ▶).
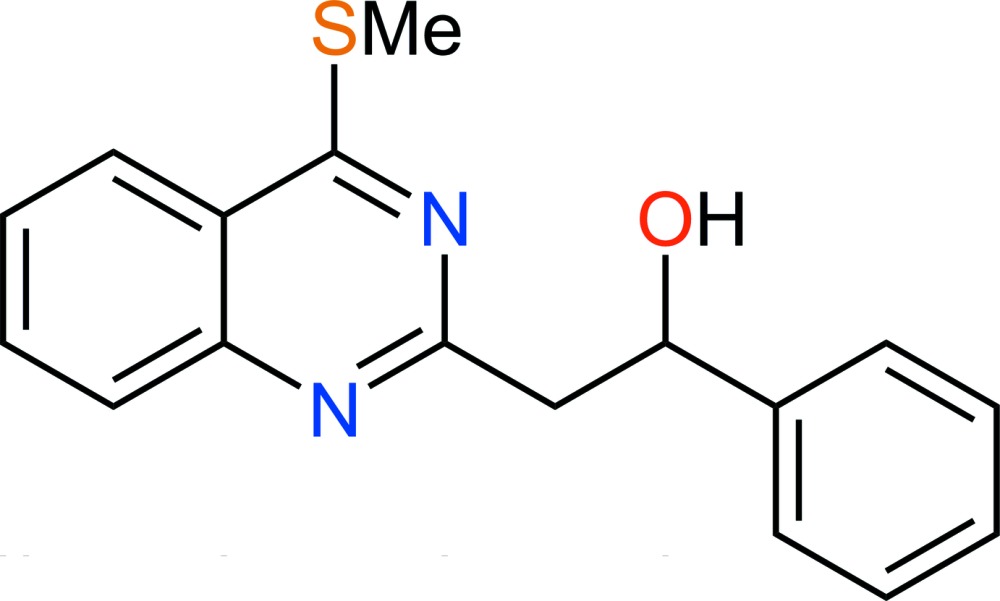


## Experimental   

### Crystal data  

C_17_H_16_N_2_OS
*M* *_r_* = 296.38
Monoclinic, 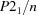

*a* = 15.6142 (3) Å
*b* = 5.6142 (1) Å
*c* = 17.2355 (3) Å
β = 101.138 (2)°
*V* = 1482.43 (5) Å^3^
*Z* = 4
Cu *K*α radiation
μ = 1.93 mm^−1^
*T* = 293 K
0.32 × 0.19 × 0.14 mm

### Data collection  

Agilent SuperNova (Dual, Cu at zero, Atlas) diffractometer
Absorption correction: multi-scan (*CrysAlis PRO*; Agilent, 2014 ▶) *T*_min_ = 0.867, *T*_max_ = 1.000
9606 measured reflections
2938 independent reflections
2688 reflections with *I* > 2σ(*I*)
*R*_int_ = 0.014

### Refinement  

*R*[*F* ^2^ > 2σ(*F* ^2^)] = 0.032
*wR*(*F* ^2^) = 0.094
*S* = 1.06
2938 reflections
192 parameters
H-atom parameters constrained
Δρ_max_ = 0.16 e Å^−3^
Δρ_min_ = −0.28 e Å^−3^

### 

Data collection: *CrysAlis PRO* (Agilent, 2014 ▶); cell refinement: *CrysAlis PRO*; data reduction: *CrysAlis PRO*; program(s) used to solve structure: *SHELXS2013* (Sheldrick, 2008 ▶); program(s) used to refine structure: *SHELXL2013* (Sheldrick, 2008 ▶); molecular graphics: *Mercury* (Macrae *et al.*, 2006 ▶), *ORTEP-3 for Windows* (Farrugia, 2012 ▶) and *CHEMDRAW Ultra* (Cambridge Soft, 2001 ▶); software used to prepare material for publication: *publCIF* (Westrip, 2010 ▶).

## Supplementary Material

Crystal structure: contains datablock(s) I, New_Global_Publ_Block. DOI: 10.1107/S1600536814019990/gg2141sup1.cif

Structure factors: contains datablock(s) I. DOI: 10.1107/S1600536814019990/gg2141Isup2.hkl

Supporting information file. DOI: 10.1107/S1600536814019990/gg2141Isup3.cml

**Fig. 1.**
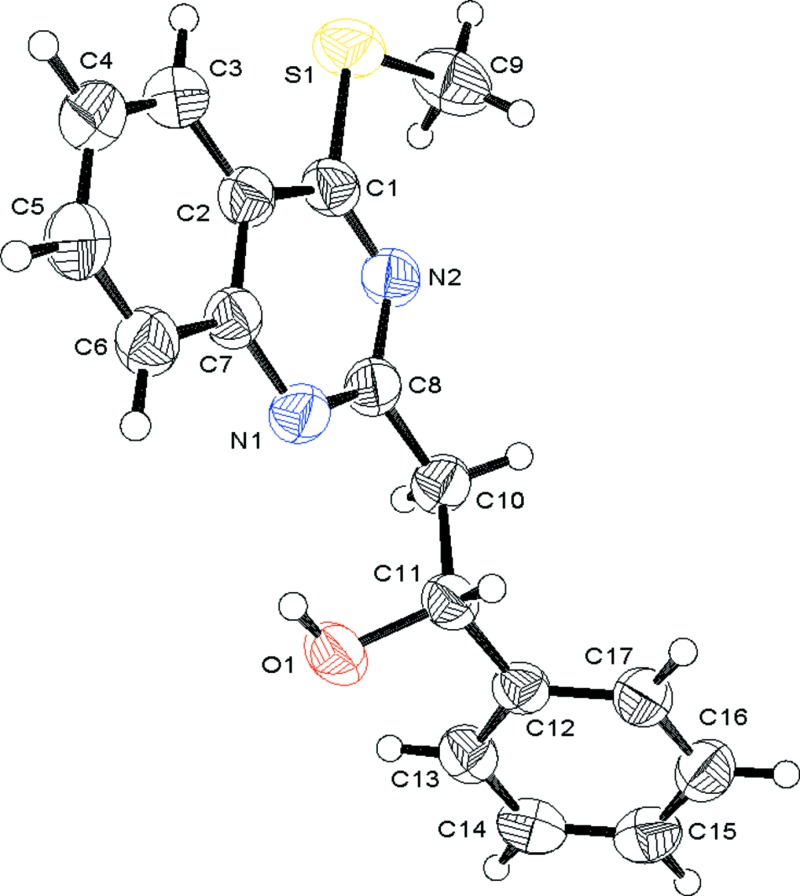
A molecule of C17H16N2OS with atom labels and 50% probability displacement ellipsoids for nonhydrogen atoms.

**Fig. 2.**
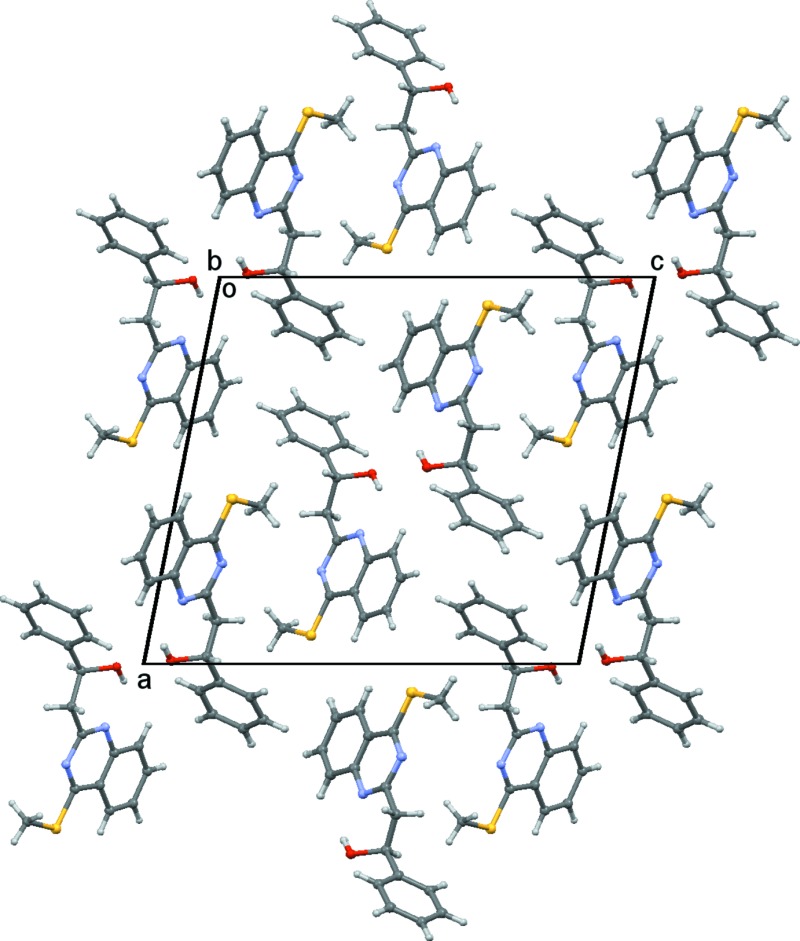
Crystal structure packing viewed down the b axis.

17 16 2 . DOI: 10.1107/S1600536814019990/gg2141fig1.tif
A mol­ecule of C_17_H_16_N_2_OS with atom labels and 50% probability displacement ellipsoids for nonhydrogen atoms.

b . DOI: 10.1107/S1600536814019990/gg2141fig2.tif
Crystal structure packing viewed down the *b* axis.

CCDC reference: 1022918

Additional supporting information:  crystallographic information; 3D view; checkCIF report

## Figures and Tables

**Table 1:** Hydrogen-bond geometry (Å, °)

*D*—H⋯*A*	*D*—H	H⋯*A*	*D*⋯*A*	*D*—H⋯*A*
O1—H1⋯N1	0.82	2.12	2.7531 (15)	134

## supplementary crystallographic information

### S1. Comment

In C_17_H_16_N_2_OS (Fig. 1), the methylsulfanylquinazoline group is planar and is oriented at an angle of 76.26 (4)° with the phenyl group. The hydroxyl group forms an intramolecular hydrogen bond to one of the quinazoline ring nitrogen atoms (Table 1) whereas the second N atom is not involved in hydrogen bonding. In the crystal structure, molecules are stacked along the *b*-axis direction (Fig. 2).

### S2. Experimental

Synthesis and crystallization: 2-(2-hydroxy-2-phenylethyl)-4-(methylsulfanyl)quinazoline was obtained in 83% yield from lithiation of 2-methyl-4-(methylsulfanyl)quinazoline with *n*-butyllithium at 78°C in anhydrous THF under nitrogen followed by reaction with benzaldehyde (Smith *et al.*, 2005*b*). Crystallization from a mixture of ethyl acetate and diethyl ether (1:3 by volume) gave the title compound as colorless crystals. The NMR and low and high resolution mass spectra for the title compound were consistent with those reported (Smith *et al.*, 2005*b*).

### S3. Refinement

H atoms were positioned geometrically and refined using a riding model with *U*_iso_(H) constrained to be 1.2 times *U*_eq_(C) except for the methyl group where it was 1.5 times with free rotation about the C—C bond. For the OH group, *U*_iso_(H) 1.5 times *U*_eq_(O) was used with free rotation about the C—O bond.

### Crystal data

**Table:**

C_17_H_16_N_2_OS	*F*(000) = 624
*M**_r_* = 296.38	*D*_x_ = 1.328 Mg m^−^^3^
Monoclinic, *P*2_1_/*n*	Cu *K*α radiation, λ = 1.54184 Å
*a* = 15.6142 (3) Å	Cell parameters from 5816 reflections
*b* = 5.6142 (1) Å	θ = 3.5–73.7°
*c* = 17.2355 (3) Å	µ = 1.93 mm^−^^1^
β = 101.138 (2)°	*T* = 293 K
*V* = 1482.43 (5) Å^3^	Block, colourless
*Z* = 4	0.32 × 0.19 × 0.14 mm

### Data collection

**Table:**

Agilent SuperNova (Dual, Cu at 0, Atlas) diffractometer	2688 reflections with *I* > 2σ(*I*)
ω scans	*R*_int_ = 0.014
Absorption correction: multi-scan (*CrysAlis PRO*; Agilent, 2014)	θ_max_ = 74.0°, θ_min_ = 3.5°
*T*_min_ = 0.867, *T*_max_ = 1.000	*h* = −19→19
9606 measured reflections	*k* = −6→6
2938 independent reflections	*l* = −11→21

### Refinement

**Table:**

Refinement on *F*^2^	0 restraints
Least-squares matrix: full	Hydrogen site location: inferred from neighbouring sites
*R*[*F*^2^ > 2σ(*F*^2^)] = 0.032	H-atom parameters constrained
*wR*(*F*^2^) = 0.094	*w* = 1/[σ^2^(*F*_o_^2^) + (0.0507*P*)^2^ + 0.2343*P*] where *P* = (*F*_o_^2^ + 2*F*_c_^2^)/3
*S* = 1.06	(Δ/σ)_max_ = 0.001
2938 reflections	Δρ_max_ = 0.16 e Å^−^^3^
192 parameters	Δρ_min_ = −0.28 e Å^−^^3^

### Special details

**Table:**

Experimental. Absorption correction: CrysAlisPro, Agilent Technologies, Version 1.171.36.28 (release 01-02-2013 CrysAlis171 .NET) (compiled Feb 1 2013,16:14:44) Empirical absorption correction in SCALE3 ABSPACK.
Geometry. All e.s.d.'s (except the e.s.d. in the dihedral angle between two l.s. planes) are estimated using the full covariance matrix. The cell e.s.d.'s are taken into account individually in the estimation of e.s.d.'s in distances, angles and torsion angles; correlations between e.s.d.'s in cell parameters are only used when they are defined by crystal symmetry. An approximate (isotropic) treatment of cell e.s.d.'s is used for estimating e.s.d.'s involving l.s. planes.

### Fractional atomic coordinates and isotropic or equivalent isotropic displacement parameters (Å^2^)

**Table:**

	*x*	*y*	*z*	*U*_iso_*/*U*_eq_	
C1	0.67633 (8)	0.0261 (2)	0.10297 (7)	0.0458 (3)	
C2	0.68632 (8)	−0.1600 (2)	0.04845 (7)	0.0450 (3)	
C3	0.62090 (9)	−0.3225 (3)	0.01440 (8)	0.0553 (3)	
H3	0.5652	−0.3110	0.0258	0.066*	
C4	0.63923 (10)	−0.4967 (3)	−0.03523 (9)	0.0601 (4)	
H4	0.5961	−0.6048	−0.0569	0.072*	
C5	0.72249 (10)	−0.5136 (3)	−0.05366 (8)	0.0569 (3)	
H5	0.7342	−0.6336	−0.0873	0.068*	
C6	0.78660 (9)	−0.3561 (3)	−0.02273 (8)	0.0523 (3)	
H6	0.8413	−0.3669	−0.0363	0.063*	
C7	0.76994 (8)	−0.1775 (2)	0.02961 (7)	0.0444 (3)	
C8	0.81859 (8)	0.1354 (2)	0.11131 (7)	0.0445 (3)	
C9	0.59215 (12)	0.3019 (3)	0.19721 (11)	0.0734 (5)	
H9A	0.6392	0.2626	0.2400	0.110*	
H9B	0.5401	0.3312	0.2176	0.110*	
H9C	0.6070	0.4421	0.1708	0.110*	
C10	0.88942 (8)	0.3057 (2)	0.14745 (8)	0.0479 (3)	
H10A	0.8870	0.3261	0.2029	0.058*	
H10B	0.8775	0.4595	0.1220	0.058*	
C11	0.98167 (8)	0.2298 (2)	0.14133 (7)	0.0429 (3)	
H11	0.9941	0.0764	0.1683	0.051*	
C12	1.04767 (8)	0.4094 (2)	0.18105 (7)	0.0414 (3)	
C13	1.06970 (9)	0.6065 (2)	0.14024 (8)	0.0483 (3)	
H13	1.0441	0.6280	0.0873	0.058*	
C14	1.12977 (9)	0.7713 (2)	0.17802 (9)	0.0538 (3)	
H14	1.1449	0.9011	0.1499	0.065*	
C15	1.16716 (9)	0.7448 (2)	0.25659 (9)	0.0544 (3)	
H15	1.2075	0.8556	0.2815	0.065*	
C16	1.14426 (9)	0.5519 (3)	0.29825 (8)	0.0541 (3)	
H16	1.1681	0.5350	0.3517	0.065*	
C17	1.08585 (8)	0.3841 (2)	0.26030 (8)	0.0477 (3)	
H17	1.0720	0.2526	0.2883	0.057*	
N1	0.83603 (7)	−0.0253 (2)	0.06177 (6)	0.0475 (3)	
N2	0.74020 (7)	0.1686 (2)	0.13399 (6)	0.0475 (3)	
O1	0.99198 (7)	0.2036 (2)	0.06187 (6)	0.0606 (3)	
H1	0.9563	0.1070	0.0393	0.091*	
S1	0.57374 (2)	0.05921 (7)	0.12865 (2)	0.06185 (14)	

### Atomic displacement parameters (Å^2^)

**Table:**

	*U*^11^	*U*^22^	*U*^33^	*U*^12^	*U*^13^	*U*^23^
C1	0.0408 (6)	0.0551 (7)	0.0426 (6)	0.0077 (5)	0.0110 (5)	0.0052 (5)
C2	0.0415 (6)	0.0531 (7)	0.0399 (6)	0.0044 (5)	0.0071 (5)	0.0040 (5)
C3	0.0463 (7)	0.0683 (9)	0.0517 (7)	−0.0049 (6)	0.0101 (6)	−0.0006 (6)
C4	0.0609 (8)	0.0649 (8)	0.0529 (8)	−0.0122 (7)	0.0070 (6)	−0.0060 (6)
C5	0.0654 (9)	0.0565 (8)	0.0487 (7)	0.0011 (7)	0.0105 (6)	−0.0077 (6)
C6	0.0504 (7)	0.0597 (8)	0.0474 (7)	0.0065 (6)	0.0107 (5)	−0.0063 (6)
C7	0.0414 (6)	0.0508 (7)	0.0405 (6)	0.0059 (5)	0.0065 (5)	0.0007 (5)
C8	0.0398 (6)	0.0514 (7)	0.0418 (6)	0.0076 (5)	0.0072 (5)	−0.0004 (5)
C9	0.0777 (11)	0.0691 (10)	0.0845 (11)	0.0061 (8)	0.0435 (9)	−0.0105 (8)
C10	0.0431 (6)	0.0510 (7)	0.0492 (7)	0.0052 (5)	0.0078 (5)	−0.0062 (5)
C11	0.0433 (6)	0.0459 (6)	0.0410 (6)	0.0028 (5)	0.0123 (5)	0.0009 (5)
C12	0.0376 (6)	0.0434 (6)	0.0451 (6)	0.0061 (5)	0.0129 (5)	−0.0006 (5)
C13	0.0508 (7)	0.0475 (7)	0.0482 (6)	0.0057 (5)	0.0139 (5)	0.0037 (5)
C14	0.0538 (7)	0.0434 (6)	0.0691 (8)	0.0010 (6)	0.0244 (6)	0.0017 (6)
C15	0.0451 (7)	0.0510 (7)	0.0677 (8)	−0.0014 (6)	0.0123 (6)	−0.0110 (6)
C16	0.0479 (7)	0.0604 (8)	0.0517 (7)	0.0045 (6)	0.0038 (6)	−0.0037 (6)
C17	0.0468 (6)	0.0489 (7)	0.0477 (6)	0.0037 (5)	0.0098 (5)	0.0039 (5)
N1	0.0390 (5)	0.0554 (6)	0.0482 (5)	0.0046 (4)	0.0084 (4)	−0.0067 (5)
N2	0.0429 (5)	0.0546 (6)	0.0462 (5)	0.0072 (5)	0.0116 (4)	−0.0021 (5)
O1	0.0655 (6)	0.0740 (7)	0.0477 (5)	−0.0177 (5)	0.0247 (4)	−0.0148 (5)
S1	0.0459 (2)	0.0755 (3)	0.0698 (2)	0.00317 (16)	0.02499 (17)	−0.00692 (17)

### Geometric parameters (Å, º)

**Table:**

C1—N2	1.3092 (17)	C9—H9C	0.9600
C1—C2	1.4340 (18)	C10—C11	1.5252 (16)
C1—S1	1.7521 (13)	C10—H10A	0.9700
C2—C7	1.4083 (17)	C10—H10B	0.9700
C2—C3	1.4100 (19)	C11—O1	1.4173 (14)
C3—C4	1.365 (2)	C11—C12	1.5082 (17)
C3—H3	0.9300	C11—H11	0.9800
C4—C5	1.400 (2)	C12—C17	1.3880 (17)
C4—H4	0.9300	C12—C13	1.3894 (18)
C5—C6	1.365 (2)	C13—C14	1.3873 (19)
C5—H5	0.9300	C13—H13	0.9300
C6—C7	1.4065 (18)	C14—C15	1.375 (2)
C6—H6	0.9300	C14—H14	0.9300
C7—N1	1.3713 (17)	C15—C16	1.384 (2)
C8—N1	1.3067 (16)	C15—H15	0.9300
C8—N2	1.3678 (16)	C16—C17	1.3843 (19)
C8—C10	1.5032 (18)	C16—H16	0.9300
C9—S1	1.7902 (17)	C17—H17	0.9300
C9—H9A	0.9600	O1—H1	0.8200
C9—H9B	0.9600		
			
N2—C1—C2	122.82 (11)	C11—C10—H10A	108.5
N2—C1—S1	119.58 (10)	C8—C10—H10B	108.5
C2—C1—S1	117.60 (10)	C11—C10—H10B	108.5
C7—C2—C3	119.24 (12)	H10A—C10—H10B	107.5
C7—C2—C1	115.14 (11)	O1—C11—C12	108.24 (10)
C3—C2—C1	125.62 (12)	O1—C11—C10	112.40 (10)
C4—C3—C2	120.08 (13)	C12—C11—C10	110.70 (10)
C4—C3—H3	120.0	O1—C11—H11	108.5
C2—C3—H3	120.0	C12—C11—H11	108.5
C3—C4—C5	120.48 (13)	C10—C11—H11	108.5
C3—C4—H4	119.8	C17—C12—C13	118.57 (12)
C5—C4—H4	119.8	C17—C12—C11	120.28 (11)
C6—C5—C4	120.70 (13)	C13—C12—C11	121.12 (11)
C6—C5—H5	119.7	C14—C13—C12	120.31 (12)
C4—C5—H5	119.7	C14—C13—H13	119.8
C5—C6—C7	120.01 (13)	C12—C13—H13	119.8
C5—C6—H6	120.0	C15—C14—C13	120.65 (13)
C7—C6—H6	120.0	C15—C14—H14	119.7
N1—C7—C6	119.03 (12)	C13—C14—H14	119.7
N1—C7—C2	121.49 (11)	C14—C15—C16	119.52 (13)
C6—C7—C2	119.47 (12)	C14—C15—H15	120.2
N1—C8—N2	126.28 (12)	C16—C15—H15	120.2
N1—C8—C10	118.73 (11)	C15—C16—C17	119.99 (13)
N2—C8—C10	114.98 (11)	C15—C16—H16	120.0
S1—C9—H9A	109.5	C17—C16—H16	120.0
S1—C9—H9B	109.5	C16—C17—C12	120.92 (12)
H9A—C9—H9B	109.5	C16—C17—H17	119.5
S1—C9—H9C	109.5	C12—C17—H17	119.5
H9A—C9—H9C	109.5	C8—N1—C7	117.30 (11)
H9B—C9—H9C	109.5	C1—N2—C8	116.95 (11)
C8—C10—C11	115.00 (10)	C11—O1—H1	109.5
C8—C10—H10A	108.5	C1—S1—C9	102.08 (7)

### Hydrogen-bond geometry (Å, º)

**Table:**

*D*—H···*A*	*D*—H	H···*A*	*D*···*A*	*D*—H···*A*
O1—H1···N1	0.82	2.12	2.7531 (15)	134
