# Supplementary figures and images for: Crystal structure of 2-[4-(methyl­sulfan­yl)quinazolin-2-yl]-1-phenyl­ethanol
Source: Acta Crystallogr Sect E Struct Rep Online. 2014 Sep 10;70(Pt 10):o1101. doi: 10.1107/S1600536814019990 (PMC4257174; doi:10.1107/S1600536814019990)

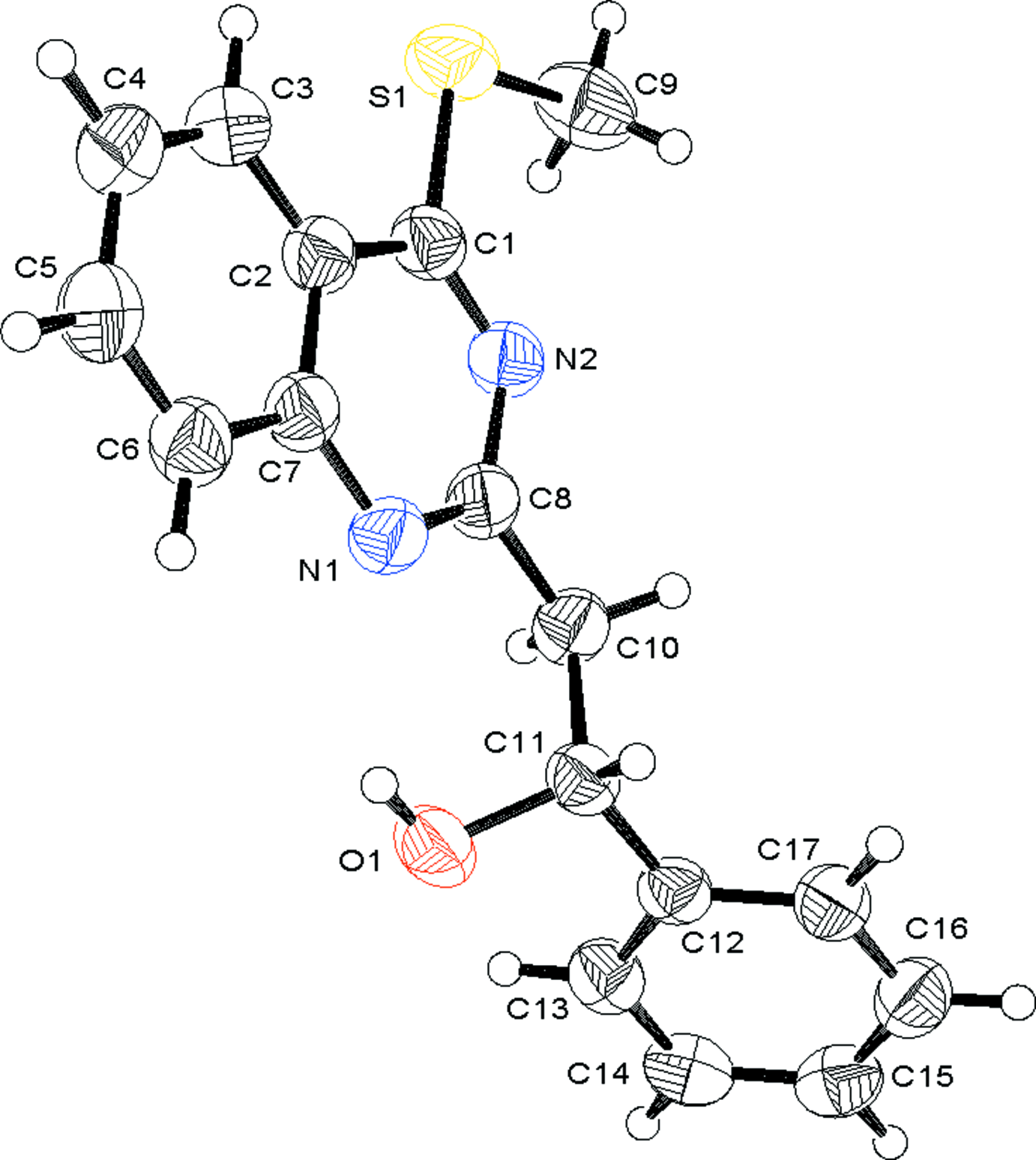

Supplement: Supplementary file 4 [file e-70-o1101-fig1.tif]

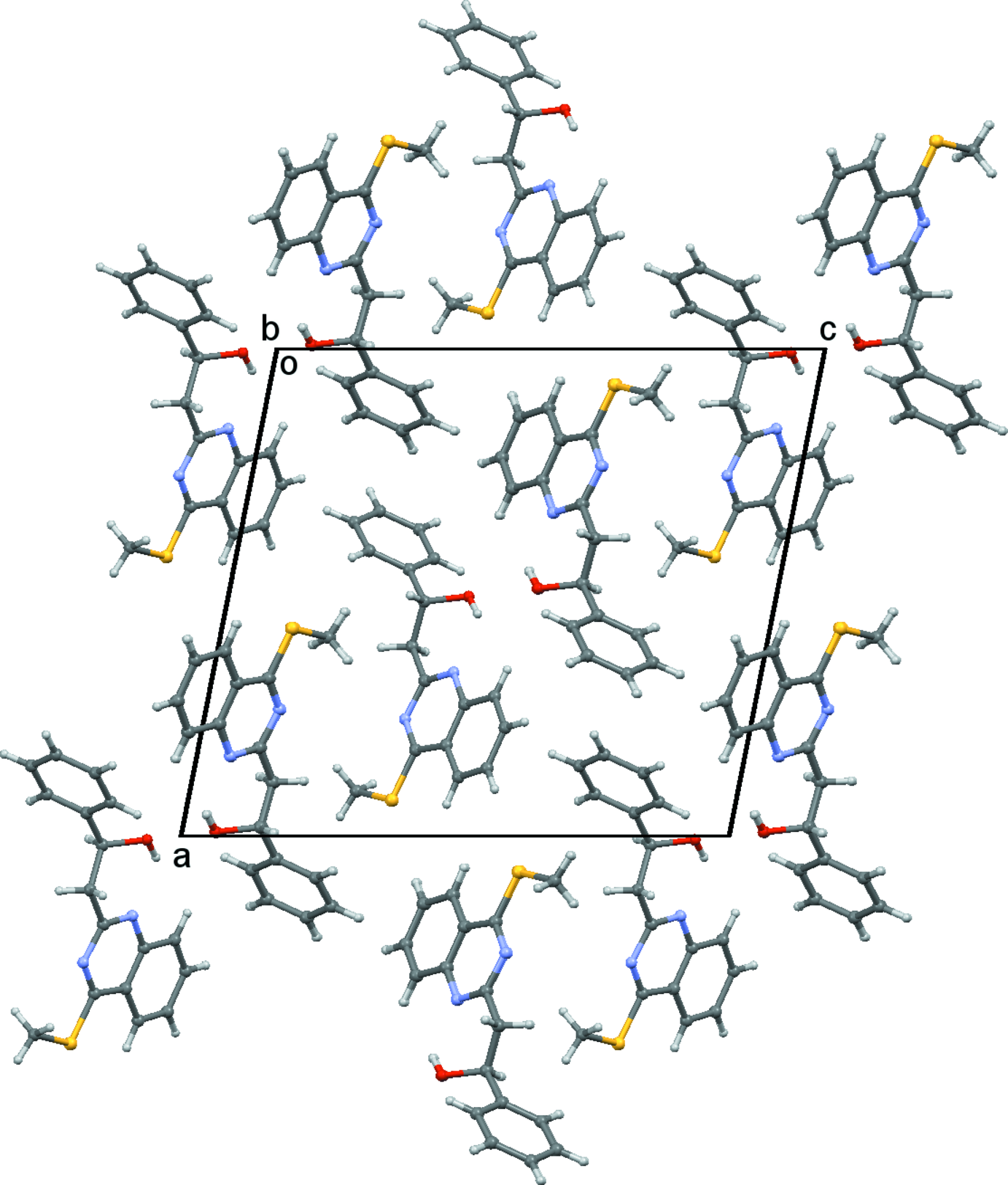

Supplement: Supplementary file 5 [file e-70-o1101-fig2.tif]
